# MicroRNA-200a/200b Modulate High Glucose-Induced Endothelial Inflammation by Targeting *O*-linked *N*-Acetylglucosamine Transferase Expression

**DOI:** 10.3389/fphys.2018.00355

**Published:** 2018-04-18

**Authors:** Wan-Yu Lo, Wen-Kai Yang, Ching-Tien Peng, Wan-Yu Pai, Huang-Joe Wang

**Affiliations:** ^1^Cardiovascular and Translational Medicine Laboratory, Department of Biotechnology, Hungkuang University, Taichung, Taiwan; ^2^Bachelor Degree Program in Animal Healthcare, Hungkuang University, Taichung, Taiwan; ^3^Department of Life Sciences, National Chung Hsing University, Taichung, Taiwan; ^4^Department of Pediatrics, Children's Hospital, China Medical University and Hospital, Taichung, Taiwan; ^5^Department of Bioscience and Biotechnology and Center of Excellence for the Oceans, National Taiwan Ocean University, Keelung, Taiwan; ^6^School of Medicine, China Medical University, Taichung, Taiwan; ^7^Cardiovascular Research Laboratory, Division of Cardiovascular Medicine, Department of Internal Medicine, China Medical University and Hospital, Taichung, Taiwan

**Keywords:** diabetes, high glucose, endothelial inflammation, *O*-linked *N*-acetylglucosamine transferase, microRNA-200a/200b

## Abstract

**Background and Aims:** Increased *O*-linked *N*-acetylglucosamine (*O*-GlcNAc) modification of proteins by *O*-GlcNAc transferase (OGT) is associated with diabetic complications. Furthermore, oxidative stress promotes endothelial inflammation during diabetes. A previous study reported that microRNA-200 (miR-200) family members are sensitive to oxidative stress. In this study, we examined whether miR-200a and miR-200b regulate high-glucose (HG)-induced OGT expression in human aortic endothelial cells (HAECs) and whether miRNA-200a/200b downregulate OGT expression to control HG-induced endothelial inflammation.

**Methods:** HAECs were stimulated with high glucose (25 mM) for 12 and 24 h. Real-time polymerase chain reaction (PCR), western blotting, THP-1 adhesion assay, bioinformatics predication, transfection of miR-200a/200b mimic or inhibitor, luciferase reporter assay, and transfection of siRNA OGT were performed. The aortic endothelium of db/db diabetic mice was evaluated by immunohistochemistry staining.

**Results:** HG upregulated OGT mRNA and protein expression and protein *O*-GlcNAcylation levels (RL2 antibody) in HAECs, and showed increased intercellular adhesion molecule 1 (ICAM-1), vascular cell adhesion molecule 1 (VCAM-1), and E-selectin gene expression; ICAM-1 expression; and THP-1 adhesion. Bioinformatics analysis revealed homologous sequences between members of the miR-200 family and the 3′-untranslated region (3′-UTR) of OGT mRNA, and real-time PCR analysis confirmed that members of miR-200 family were significantly decreased in HG-stimulated HAECs. This suggests the presence of an impaired feedback restraint on HG-induced endothelial protein *O*-GlcNAcylation levels because of OGT upregulation. A luciferase reporter assay demonstrated that miR-200a/200b mimics bind to the 3′-UTR of OGT mRNA. Transfection with miR-200a/200b mimics significantly inhibited HG-induced OGT mRNA expression, OGT protein expression; protein *O*-GlcNAcylation levels; ICAM-1, VCAM-1, and E-selectin gene expression; ICAM-1 expression; and THP-1 adhesion. Additionally, siRNA-mediated OGT depletion reduced HG-induced protein *O*-GlcNAcylation; ICAM-1, VCAM-1, and E-selectin gene expression; ICAM-1 expression; and THP-1 adhesion, confirming that HG-induced endothelial inflammation is partially mediated via OGT-induced protein *O*-GlcNAcylation. These results were validated *in vivo*: tail-vein injection of miR-200a/200b mimics downregulated endothelial OGT and ICAM-1 expression in db/db mice.

**Conclusion:** miR-200a/200b are involved in modulating HG-induced endothelial inflammation by regulating OGT-mediated protein *O*-GlcNAcylation, suggesting the therapeutic role of miR-200a/200b on vascular complications in diabetes.

## Introduction

Diabetic vascular disease—a low-grade, chronic inflammatory disease—is a major cause of death in developed countries. Increased levels of reactive oxygen species (ROS) in endothelial cells are involved in the pathophysiology of diabetic vascular complications by activating downstream pathways, leading to increases in polyol pathway flux, advanced glycation end product formation, advanced glycation end product receptor formation, protein kinase C isoform activation, and hexosamine biosynthetic pathway (HBP) flux (Giacco and Brownlee, 2010). Hyperglycemia is the primary abnormality causing diabetic endothelial dysfunction (Nakagami et al., 2005). Normally, glucose enters cells and is metabolized into glucose-6-phosphate and fructose-6-phosphate, the latter of the two being typically destined for the glycolytic pathway. Only a small amount of fructose-6-phosphate (2–5%) is converted to glucosamine-6-phosphate by glutamine:fructose 6-phosphate amidotransferase. This conversion commits fructose-6-phosphate to entering the HBP, which produces UDP-*N*-acetylglucosamine (UDP-GlcNAc) as an important sensor of nutrition flux by integrating the metabolic processes of glucose, fatty acids, amino acids, and nucleotides (Bond and Hanover, 2015).

*O*-GlcNAc glycosylation (*O*-GlcNAcylation) is an important post-translational modification of nuclear, cytoplasmic, and mitochondrial proteins which modulates the stability, subcellular localization, and activity of proteins (Issad et al., 2010). *O*-GlcNAcylation modification is controlled by two enzymes: β-*N*-acetylglucosaminidase (OGA), which removes *O*-linked *N*-acetylglucosamine (*O*-GlcNAc) from *O*-GlcNAcylated proteins, and *O*-GlcNAc transferase (OGT), which adds *O*-GlcNAc from UDP-GlcNAc to the threonine and serine residues of targeted proteins (Bond and Hanover, 2015). More than 4000 *O*-GlcNAcylated protein targets have been identified; these proteins play a key role in multiple biological processes, including transcription, translation, proteasomal degradation, homeostasis, epigenetic regulation, and stress responses (Ma and Hart, 2014). Therefore, aberrant *O*-GlcNAcylation has been linked to the pathophysiology of several chronic diseases, including cancer, neurodegeneration, and type 2 diabetes (Bond and Hanover, 2013). Increased oxidative stress is closely linked to augmented *O*-GlcNAcylation in diabetes (Giacco and Brownlee, 2010). In contrast, increased activation of HBP with high-glucose (HG) and glucosamine has been reported to induce mesangial ROS production (Singh et al., 2007). Furthermore, *O*-GlcNAcylation depletion by shRNA-mediated downregulation of OGT has been shown to prevent HG-induced mesangial ROS production (Goldberg et al., 2011). These findings indicate significant interplay between redox signaling and *O*-GlcNAcylation modification in diabetes (Lima et al., 2012).

MicroRNAs (miRs) play an essential role in mediating the post-transcriptional regulation of the endothelial oxidative response (Marin et al., 2013). An increasing number of studies has suggested that multiple miRs are involved in the pathogenesis of diabetes (Shantikumar et al., 2012). Particularly, members of the miR-200 family are highly sensitive to ROS. Magenta and colleagues reported that miR-200 family members were increased in H_2_O_2_-stimulated human umbilical endothelial cells, which play a critical role in ROS-mediated senescence and apoptosis by downregulating the zinc-finger E-box binding homeobox 1 (ZEB-1) (Magenta et al., 2011). The five known miR-200 members are located on two chromosomes (chromosome 12: miR-141 and miR-200c; chromosome 1, miR-200a, miR-200b, and miR-429) and are highly conserved in higher vertebrates (Gheldof et al., 2012). Moreover, they can be separated into two functional groups that differ in seed sequences by only a single nucleotide (underlined). The seed sequence 5′-AACACUG-3′ defines functional group 1 (miR-141/200a), while 5′-AAUACUG-3′ defines functional group 2 (miR-200b/200c/429) (Senfter et al., 2016). The miR-200 family is now known to play an inhibitory role in cancer progression because of the strong suppressive effects of its members on cell transformation, migration, proliferation, invasion, and metastasis (Humphries and Yang, 2015). However, reports of miR-200 family members in diabetes-associated studies are conflicting. Some studies showed that miR-200 family members have harmful effects on diabetes progression (Filios et al., 2014; Belgardt et al., 2015), diabetes-induced inflammation (Reddy et al., 2012), and diabetes-induced endothelial dysfunction (Zhang et al., 2016), while others showed that members of the miR-200 family have protective effects because of their anti-inflammatory (McArthur et al., 2011) and anti-fibrotic properties (Wei et al., 2014).

Although ROS is involved in modulating the miR-200 family and protein *O*-GlcNAcylation, the interplay between these factors is not clear. In this study, we investigated the role of miR-200 members in regulating endothelial inflammation mediated by OGT-induced *O*-GlcNAcylation in HG-treated human aortic endothelial cells (HAECs) and examined the aortic endothelial tissues in the miR-200a/200b mimics-treated db/db type 2 diabetic mice used as an *in vivo* model.

## Materials and methods

### Endothelial cell culture

HAECs were purchased from Cell Applications, Inc. (San Diego, CA, USA) and cultured in endothelial cell growth medium (Cell Applications, Inc.) as previously described (Wang et al., 2014). Most of the chemicals were purchased from Sigma-Aldrich (St. Louis, MO, USA) unless otherwise specified. High-glucose (25 mM) stimulation for 12 and 24 h of HAECs was performed in different experiments. L-Glucose solutions (LG, 25 mM) were used as the osmotic control. THP-1 cells (i.e., a human monocytic cell line) were obtained from the American Type Culture Collection (Manassas, VA, USA) and maintained as previously described (Wang et al., 2014).

### Real-time polymerase chain reaction (PCR)

The mRNA expression levels of genes of interest in HAECs were analyzed using a StepOnePlus Real-Time PCR instrument (Applied Biosystems, Foster, CA, USA) (Bianconcini et al., 2009; Wang and Lo, 2017). Gene expression levels were analyzed by StepOne software v2.2, and glyceraldehyde-3-phosphate dehydrogenase (GAPDH) was used as an internal control. The primer sequences for GAPDH, OGT, OGA, vascular cell adhesion protein 1 (VCAM-1), intercellular adhesion molecule 1 (ICAM-1), E-selectin, and ZEB-1 are provided in Supplemental Data 1.

### Western blotting

Each protein sample (40 μg) was separated by sodium dodecyl sulfate polyacrylamide gel electrophoresis and transferred onto a polyvinylidene fluoride membrane using semidry transfer equipment, as previously described (Pai et al., 2017). Protein expression levels were normalized to that of GAPDH expression. Primary antibodies against GAPDH (1:5000, Santa Cruz Biotechnology, Dallas, TX, USA), OGT (1:1000; Abcam, Cambridge, UK), *O*-GlcNAcylation (RL2, 1:1000; Abcam), and ICAM-1 (1:1000; Cell Signaling Technology, Danvers, MA, USA) were used. Immunostaining was performed using SuperSignal West Pico Chemiluminescent Substrate (Thermo Fisher, Waltham, MA, USA) for OGT, and *O*-GlcNAcylation (RL2), and SuperSignal West Femto Maximum Sensitivity Substrate (Thermo Fisher) was used for ICAM-1 (Wang and Lo, 2017).

### Monocyte adhesion assay

In adhesion experiments, the THP-1 cells were stained with calcein acetoxymethyl ester (Calcein-AM; Molecular Probes, Eugene, OR, USA), as previously described (Wang et al., 2014; Lo et al., 2017). Briefly, the THP-1 cells were labeled with the dye (7.5 μM) for 30 min before performing the adhesion assay. The HAECs (10^5^ cells/well) were stimulated with HG for 24 h and incubated with labeled THP-1 cells (the ratio of THP-1: HAECs = 1:7) for 10 min. Non-adherent THP-1 cells were washed away with phosphate-buffered saline (PBS). All adherent THP-1 cells were counted in 10 randomly-selected fields using a fluorescent microscope (Olympus, Tokyo, Japan).

### TaqMan miR assay for members of the miR-200 family

Total RNA, including miRs, were extracted from the HAECs using a High Pure miRNA Isolation Kit (Invitrogen, Carlsbad, CA, USA). Variable miRs and RNU6B-specific cDNA were synthesized according to the TaqMan microRNA assay kit (RNU6B: Cat No. 602003; miR-200a: Cat No. P160705-000 H12; miR-141: Cat No. P160621-009 F07; miR-200b: Cat No. P160801-000 H20; miR-200c: Cat No. P160808-004 G12; miR-429: Cat No. P160720-008 G12) protocol (Applied Biosystems). RNU6B was used as an internal control. Quantitative real-time PCR was performed using a StepOnePlus Real-Time PCR instrument (Applied Biosystems).

### Transfection of miR-200a and miR-200b mimics

miR-200a mimic, miR-200b mimic, miR-200a inhibitor, miR-200b inhibitor, and negative control (NC) were transfected into the HAECs as previously described (Wang et al., 2014; Lo et al., 2017). After transfection, the HAECs were treated with high levels of glucose for 24 h, after which the expression levels of OGT mRNA, OGT, protein *O*-GlcNAcylation, ICAM-1 mRNA, VCAM-1 mRNA, E-selectin mRNA, and ICAM-1 were analyzed. THP-1 monocyte adhesion assays were performed after miR-200a mimic and miR-200b mimic transfection.

### Luciferase reporter assay

A segment of the OGT 3′-untranslated region (UTR) that includes both miR-200a and miR-200b binding sites was constructed into the pmirGLO vector (Promega, Madison, WI, USA). The sequence is listed in Supplemental Data 2. HAECs were cotransfected with 1 μg of constructed plasmids and 100 nM of miR-200a mimic, miR-200b mimic, or NC using Lipofectamine™ 2000 (Invitrogen). After 24 h of transfection, the cells were harvested to determine luciferase activity using the Luciferase Assay System Kit (Promega, E1500), as previously described (Lo et al., 2017).

### OGT gene silencing

For OGT gene knockdown, the HAECs were transfected with 100 nM human OGT siRNA (GeneDirex, Keelung, Taiwan), as previously described (Wang et al., 2016; Pai et al., 2017). Scrambled NC siRNA was included as a negative control. After OGT siRNA transfection, the medium was changed to fresh endothelial cell growth medium, and the HAECs were stimulated with HG for 24 h. After HG treatment, the expression levels of OGT mRNA, OGT, protein *O*-GlcNAcylation, ICAM-1 mRNA, VCAM-1 mRNA, E-selectin mRNA, and ICAM-1 were determined. THP-1 monocyte adhesion assays were performed after OGT siRNA transfection.

### Type 2 db/db diabetic mouse model experiments

Animal studies were approved by the Hungkuang University Institutional Animal Care and Use Committee with a permit Number: HK 10607. Male db/db diabetic mice were obtained from the National Laboratory Animal Center (Nangang, Taipei, Taiwan). Fourteen-week-old db/db mice were injected with 100 μL miR-200a mimic, miR-200b mimic, or NC (13 μg per week, three times) by tail-vein injection using equal volume mixtures of Lipofectamine™ 2000 and miR-200a mimic, miR-200b mimic, or NC. The non-diabetic control db/m group was administered with equal volume mixtures of vehicle and PBS. Mice were sacrificed by CO_2_ narcosis after 3 weeks. Aortic tissue was cautiously excised and fixed with formalin solution. Paraffin sections of the aorta were prepared for immunohistochemistry (IHC) staining.

### IHC staining

IHC staining was performed as previously described (Yang et al., 2016). Briefly, after deparaffinization and rehydration, the aortic sections were rinsed with PBS and then pre-incubated with sodium citrate buffer (10 mM, pH 6.0) at 95°C for 10 min. After cooling and washing with PBS, endogenous peroxidase was inactivated by incubating the sections with 3% hydrogen peroxide for 10 min. Next, the sections were incubated with anti-OGT (ab96718; abcam; dilution 1:100) or anti-ICAM-1 (MA5407; Thermo Fisher; dilution 1:100) at 4°C overnight before being processed with a commercial kit (PicTure™; Zymed, South San Francisco, CA, USA). The sections were counterstained with hematoxylin (Cat. No.1.05175.0500; Merck, Darmstadt, Germany) for histological evaluation. The sections were covered with a coverslip using mounting solution (#00-8110; Thermo Fisher). Finally, the immunosignals were examined under a microscope (BX50; Olympus) equipped with the cooling-charge-coupled-device camera (DP72; Olympus) using CellSens software (standard v1.4; Olympus). Immunoreactivity signals observed in the endothelial layers of the db/m (vehicle), db/db (negative control), db/db (miR-200a mimic), and db/db (miR-200b mimic) groups were quantified as previously described (Federici et al., 2002).

### Statistical analysis

Statistical analyses were performed using the SPSS 12.0 statistical software (SPSS Inc., Chicago, IL, USA). All data are presented as the mean ± standard error of the mean (SEM). Pair-wise comparisons were carried out using Student's *t*-test. Three or more groups were compared by one-way analysis of variance with Tukey *post-hoc* tests. Significant differences were defined as *p* < 0.05.

## Results

### HG increases OGT expression, protein *O*-GlcNAcylation, and inflammation in the HAECs

**Figure 1:**
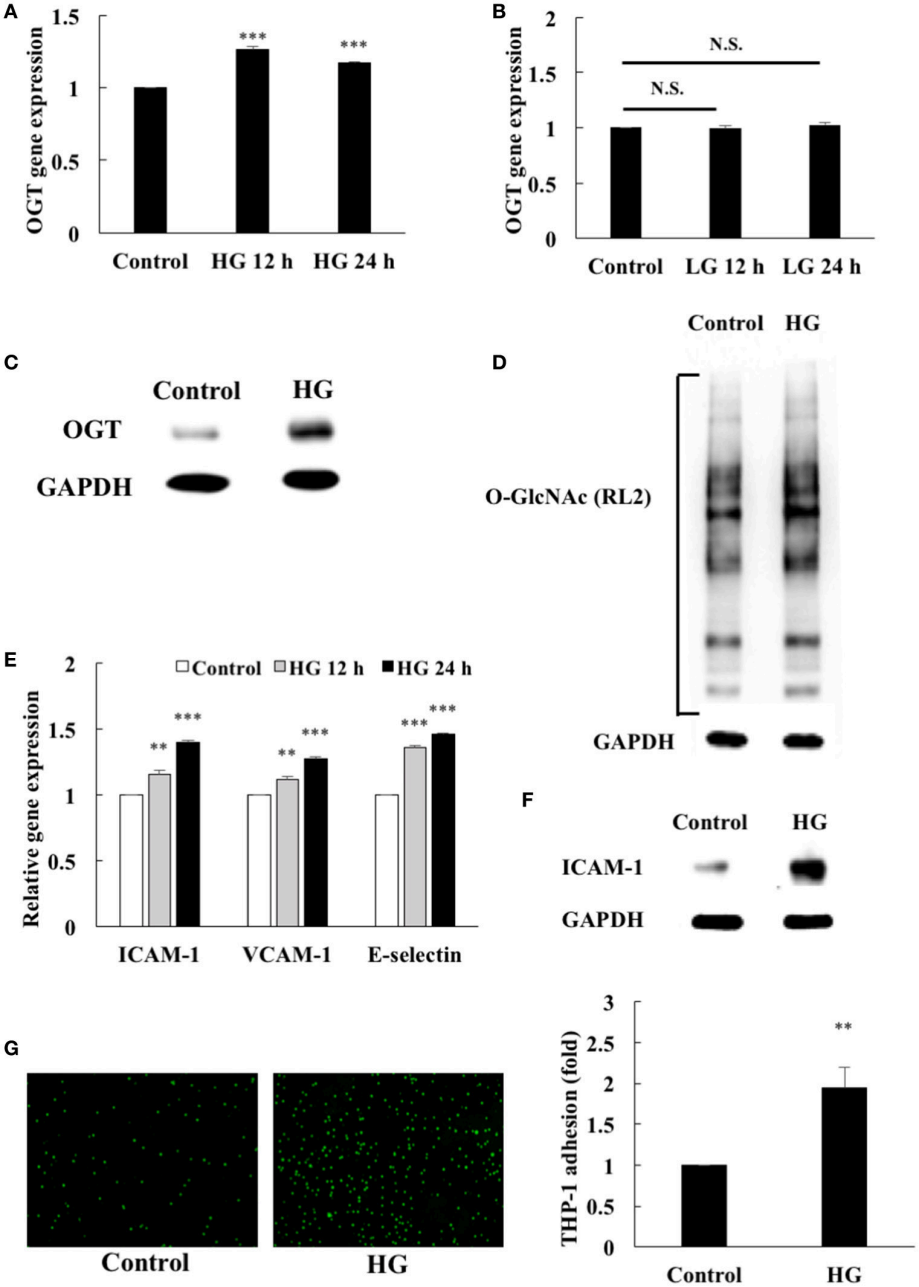
Endothelial OGT expression, protein *O*-GlcNAcylation, and inflammatory phenotypes were induced by high glucose (HG). **(A)** HAECs were stimulated with HG (25 mM) for 12 and 24 h. Real-time PCR revealed that HG stimulation induced 1.27- and 1.17-fold increases, respectively, in OGT mRNA levels *N* = 5. ***p < 0.001 compared with control. **(B)** L-Glucose (LG) (25 mM) did not modulate OGT mRNA levels *N* = 4. N.S. not significant. **(C)** Stimulation of the HAECs with HG for 24 h induced a significant increase in OGT expression. The blot is representative of three independent experiments. **(D)** Stimulation of the HAECs with HG for 24 h induced a significant increase in protein *O*-GlcNAcylation as detected using the RL2 antibody. The blot is representative of three independent experiments. **(E)** Stimulation of the HAECs with HG for 12 and 24 h induced 1.15- and 1.40-fold increases, 1.11- and 1.27-fold increases, and 1.36- and 1.46-fold increases in ICAM-1, VCAM-1, and E-selectin gene expression levels, respectively *N* = 5. **p < 0.01 and ***p < 0.001 compared with control. **(F)** Stimulation of the HAECs with HG for 24 h induced a significant increase in ICAM-1 expression. The blot is representative of three independent experiments. **(G)** Stimulation of the HAECs with HG for 24 h induced a 1.95-fold increase in THP-1 adhesion to HAECs *N* = 4. **p < 0.01 compared with control.

First, we examined the effects of HG stimulation on endothelial OGT expression. After 12 and 24 h treatment, HG stimulation caused significant (1.27- and 1.17-fold, respectively) increases in OGT gene expression in the HEACs compared with that in the unstimulated control (Figure 1A). Similar to its stimulatory effect on OGT gene expression, HG treatment for 12 and 24 h also caused significant increases (1.19- and 1.28-fold, respectively) in OGA gene expression compared with that in the unstimulated control (Supplemental Data 3). In contrast, the osmotic control LG did not modulate the gene expression levels of OGT (Figure 1B). The expression of OGT protein (Figure 1C) were also significantly increased after 24 h of HG stimulation (Figure 1C) and was associated with increased protein *O*-GlcNAcylation (Figure 1D). Monocyte adhesion to the endothelium using cellular adhesion molecules (ICAM-1, VCAM-1, E-selectin) initiates endothelial inflammation. HG stimulation for 12 and 24 h caused 1.15- and 1.40-fold increases in ICAM-1 gene expression, respectively (Figure 1E). Additionally, 12- and 24-h HG stimulation caused 1.11- and 1.27-fold increases in VCAM-1 gene expression and 1.36- and 1.46-fold increases in E-selectin gene expression, respectively (Figure 1E). The expression of ICAM-1 was also significantly upregulated after 24 h HG stimulation (Figure 1F) and was associated with a 1.95-fold increase in the adhesion of the THP-1 cells to the HAECs (Figure 1G).

### OGT is a direct target of miR-200a and miR-200b

**Figure 2:**
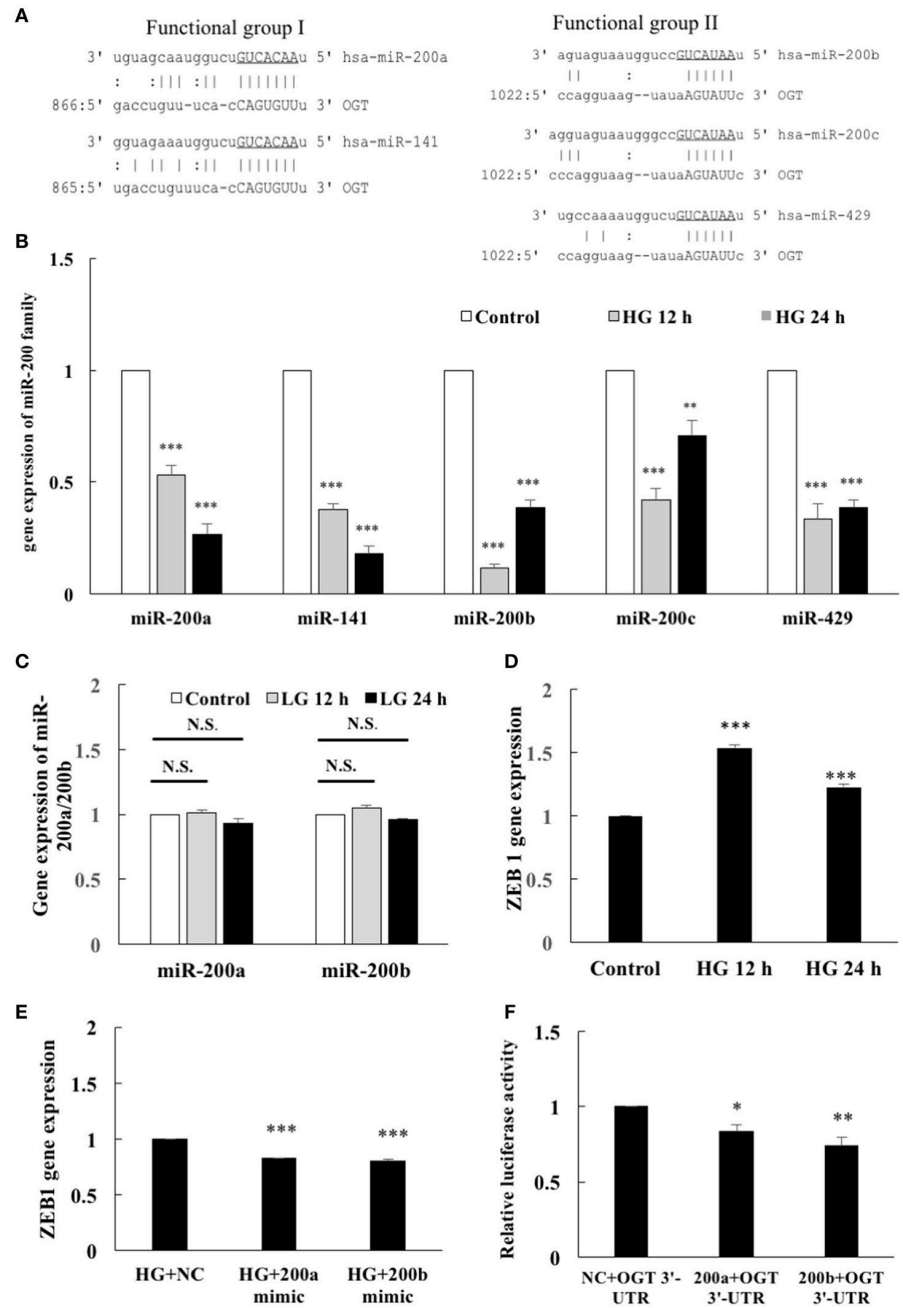
OGT is a direct target of miR-200a and miR-200b. **(A)** Bioinformatics miR target analysis identified homologies between miR-200a, miR-141, miR-200b, miR-200c, miR-429, and the 3′-UTR of human OGT mRNA, indicating potential regulation of OGT by these miRs. | AU or CG.: GU wobble. **(B)** Real-time PCR showed that HG stimulation for 12 h decreased miR-200a, miR-141, miR-200b, miR-200c, and miR-429 expression levels to 53, 38, 11, 42, and 33% of the control level, respectively. Similarly, 24 h HG stimulation decreased miR-200a, miR-141, miR-200b, miR-200c, and miR-429 expression levels to 27, 18, 39, 71, and 39% of the control level, respectively *N* = 5. **p < 0.01 and ***p < 0.001 compared with control. miR expression levels were normalized to U6 expression. **(C)** LG did not modulate miR-200a/200b expression levels *N* = 4. N.S. not significant. **(D)** HAECs were stimulated with HG (25 mM) for 12 and 24 h. Real-time PCR demonstrates that stimulation of HAECs with HG induced 1.53-fold (12 h) and 1.23-fold (24 h) increases in ZEB1 mRNA levels *N* = 5. ***p < 0.001 compared with control. **(E)** miR-200a/200b mimic transfection inhibited HG-induced endothelial ZEB1 mRNA expression to 83 and 80% of the control level, respectively, in the HG-stimulated HAECs *N* = 4. ***p < 0.001 compared with the HG-treated HAECs transfected with a negative control (HG+NC). **(F)** A luciferase reporter assay showed that cotransfection of pmirGLO-OGT-3′-UTR and the miR-200a/200b mimics downregulated the luciferase signal to 83 and 74%, respectively, of the control luciferase signal, respectively *N* = 5. *p < 0.05 and **p < 0.01 compared with the negative control.

To identify possible binding partners of the 3′-UTR of human OGT mRNA, *in silico* analyses using miRanda-mirSVR (www.microrna.org) and miRDB (www.mirdb.org) database with the help of manual sequence alignment were performed. All members of the miR-200 family were showed homology with the 3′-UTR of human OGT mRNA in their seed sequences (Figure 2A), indicating potential regulation of OGT by miR-200 members. Positions 2–8 of the seed sequence of the functional group 1 (5′-AACACUG-3′), and the positions 2–7 of the functional group 2 (5′-AAUACU-3′) were predicated to bind to the 3′-UTR of OGT mRNA. As shown in Figure 2B, real-time PCR analyses showed that HG stimulation for 12 h resulted in a significant decrease in miR-200a, miR-141, miR-200b, miR-200c, and miR-429 expression levels to 53, 38, 11, 42, and 33%, respectively, of the levels in the unstimulated control. Similarly, HG stimulation for 24 h caused a significant decrease in the miR-200a, miR-141, miR-200b, miR-200c, and miR-429 expression levels to 27, 18, 39, 71, and 39%, respectively, of the levels in the unstimulated control. Real-time PCR data revealed that the most highly expressed miRs among functional groups 1 and 2 were miR-200a and miR-200b, respectively. Therefore, miR-200a and miR-200b were selected for further analysis. Although HG caused a significant decrease in the miR-200a and miR-200b expression levels, the osmotic control LG did not modulate the expression levels of miR-200a and miR-200b (Figure 2C). The transcriptional inhibitor ZEB-1 is a well-known target of the miR-200 family members through a reciprocal repression mechanism to promote epithelial-mesenchymal transition and cancer invasion (Burk et al., 2008). To determine whether HG-induced downregulation of miR-200 members also modulates ZEB-1 gene expression, we examined the effects of HG on ZEB-1 expression. After 12- and 24-h stimulation, HG caused significant (1.54- and 1.23-fold, respectively) increases in ZEB-1 gene expression in the HEACs compared with that in the in unstimulated control cells (Figure 2D). Moreover, miR-200a/200b mimics significantly decreased the ZEB-1 gene expression levels to 83 and 80%, respectively, of the expression in the negative control group after 24 h HG-stimulation in the HAECs (Figure 2E). These results confirm that ZEB-1 is a target of miR-200a/200b. Furthermore, downregulation of miR-200 family members in the HG-stimulated HAECs can modulate the expression levels of ZEB-1. To investigate whether miR-200a/200b can interact with the 3′-UTR of OGT mRNA, we performed a luciferase reporter assay. As shown in Figure 2F, cotransfection of pmirGLO-OGT-3′-UTR and either the miR-200a or miR-200b mimic resulted in a decrease in the luciferase signal to 83 and 74%, respectively, of that in the negative control, confirming direct binding of miR-200a/200b to the 3′-UTR of OGT mRNA.

### miR-200a/200b mimics inhibit HG-induced endothelial OGT expression, protein *O*-GlcNAcylation, and inflammatory phenotypes

**Figure 3:**
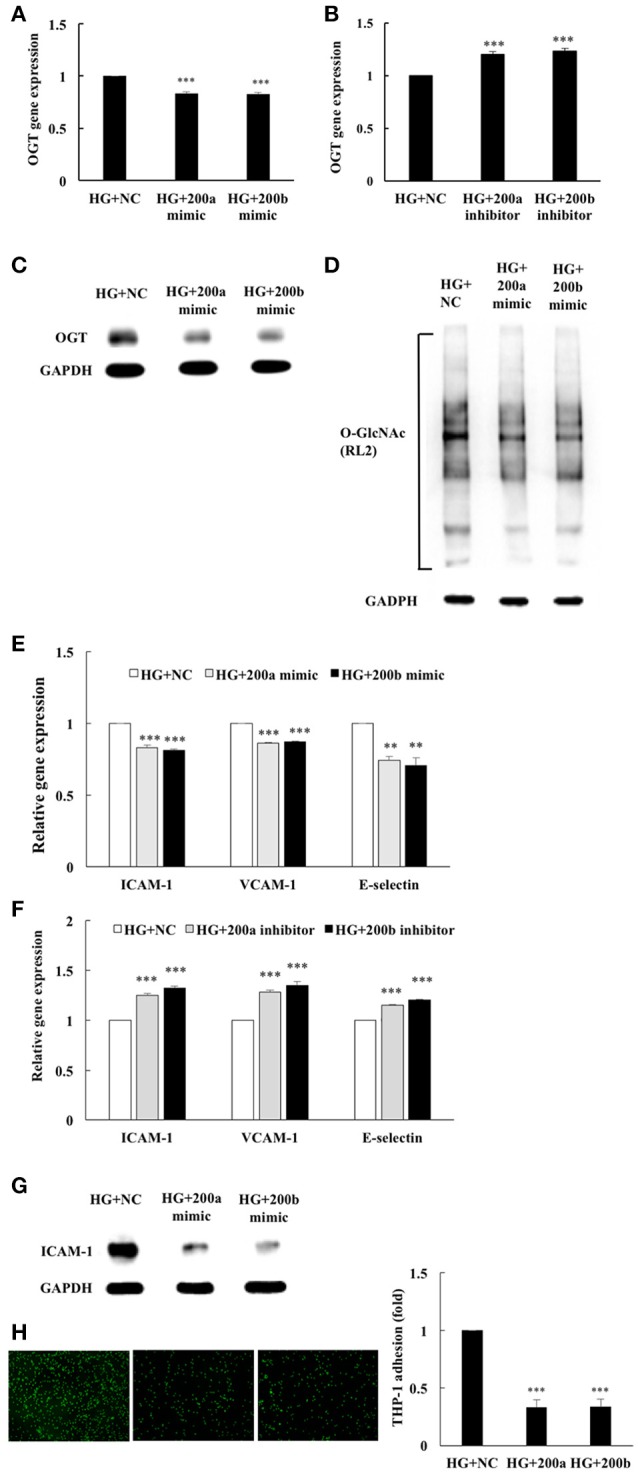
miR-200a/200b mimics inhibited HG-induced endothelial OGT expression, protein *O*-GlcNAcylation, and inflammatory phenotypes. **(A)** Transfection of miR-200a/200b mimics inhibited HG-induced endothelial OGT mRNA expression to 83 and 82% of the control level, respectively *N* = 4. ***p < 0.001 compared with the HG-treated HAECs transfected with a negative control (HG+NC). **(B)** Transfection of miR-200a/200b inhibitors enhanced HG-induced endothelial OGT mRNA expression to 1.20- and 1.23-fold of the control level, respectively *N* = 4. ***p < 0.001 compared with HG+NC. **(C)** The stimulatory effect of HG on OGT protein expression was significantly lower in the miR-200a/200b mimic-transfected HAECs than in the HAECs transfected with negative control (HG+NC). The blot is representative of three independent experiments. **(D)** The stimulatory effect of HG on protein *O*-GlcNAcylation was significantly inhibited in miR-200a/200b mimic-transfected HAECs compared to HG+NC. The blot is representative of three independent experiments. **(E)** Transfection of miR-200a/200b mimics inhibited HG-induced endothelial ICAM-1 mRNA expression to 83 and 81%, VCAM-1 mRNA expression to 86 and 87%, and E-selectin mRNA expression to 74 and 70% of the control level, respectively *N* = 4. **p < 0.01 and ***p < 0.001 compared with HG+NC. **(F)** Transfection of miR-200a/200b inhibitors enhanced HG-induced endothelial ICAM-1 mRNA expression by 1.25- and 1.32-fold, VCAM-1 mRNA expression by 1.28- and 1.35-fold, and E-selectin mRNA expression by 1.15-fold and 1.20-fold of the control level *N* = 4. ***p < 0.001 compared with HG+NC. **(G)** The stimulatory effect of HG on ICAM-1 expression was significantly inhibited in miR-200a/200b mimic-transfected HAECs compared with HG+NC. The blot is representative of three independent experiments. **(H)** miR-200a/200b mimics inhibited HG-induced THP-1 adhesion to 33 and 34% of the control level, respectively *N* = 4. ***p < 0.001 compared with HG+NC.

To examine the potential regulation of OGT expression by the miR-200 family, transfection assays with 200a/200b mimics or inhibitors were performed to determine whether miR-200a/200b mimics modulates OGT gene expression. As shown in Figure 3A, transfection of miR-200a/200b mimics in HG-stimulated HAECs caused a significant decrease in the OGT gene expression level to 83 and 82%, respectively, of that in the negative control (Figure 3A). Interestingly, transfection of miR-200a mimics in the HG-stimulated HAECs caused a non-significant decrease in OGA expression, to 95% (*p* = 0.089) of that in the negative control. Transfection of 200b mimics in the HG-stimulated HAECs caused a significant decrease in OGA expression, to 91% (*p* = 0.007) of that in the negative control (Supplemental Data 4). In contrast, transfection of miR-200a/200b inhibitors in the HG-stimulated HAECs caused a significant increase in OGT gene expression level, by 1.20- and 1.23-fold, respectively, of that in the negative control (Figure 3B). Transfection of miR-200a/200b mimics also significantly downregulated OGT protein expression (Figure 3C) and was associated with decreased protein *O*-GlcNAcylation in the HG-stimulated HAECs as compared with the negative control group (Figure 3D). To determine whether reduced OGT and protein *O*-GlcNAcylation expression are associated with reduced endothelial inflammation in the miR-200a/200b mimics-transfected HG-stimulated HAECs, we measured ICAM-1, VCAM-1, and E-selectin gene expression levels, ICAM-1 expression, and THP-1 monocyte adhesion. As shown in Figure 3E, transfection of miR-200a/200b mimics respectively inhibited HG-induced endothelial ICAM-1 mRNA expression to 83 and 81%, VCAM-1 mRNA expression to 86 and 87%, and E-selectin mRNA expression to 74 and 70%, of the negative control expression levels. In contrast, transfection of miR-200a/200b inhibitors respectively increased HG-induced endothelial ICAM-1 mRNA expression to 1.25- and 1.32-fold, VCAM-1 mRNA expression to 1.28- and 1.35-fold, and E-selectin mRNA expression to 1.15-fold and 1.20-fold, of the negative control expression levels (Figure 3F). ICAM-1 expression was also significantly decreased by transfection with miR-200a/200b mimics in the HG-stimulated HAECs (Figure 3G) and was associated with decreased adhesion of the THP-1 cells to the HAECs, to 33 and 34%, respectively, of the adhesion levels in the negative control group (Figure 3H). These results indicate that miR-200a/200b mimics possess anti-inflammatory properties in HG-induced endothelial inflammation, possibly because of their ability to modulate protein *O*-GlcNAcylation levels via OGT downregulation.

### OGT depletion by siRNA inhibits HG-induced endothelial protein *O*-GlcNAcylation and inflammation

**Figure 4:**
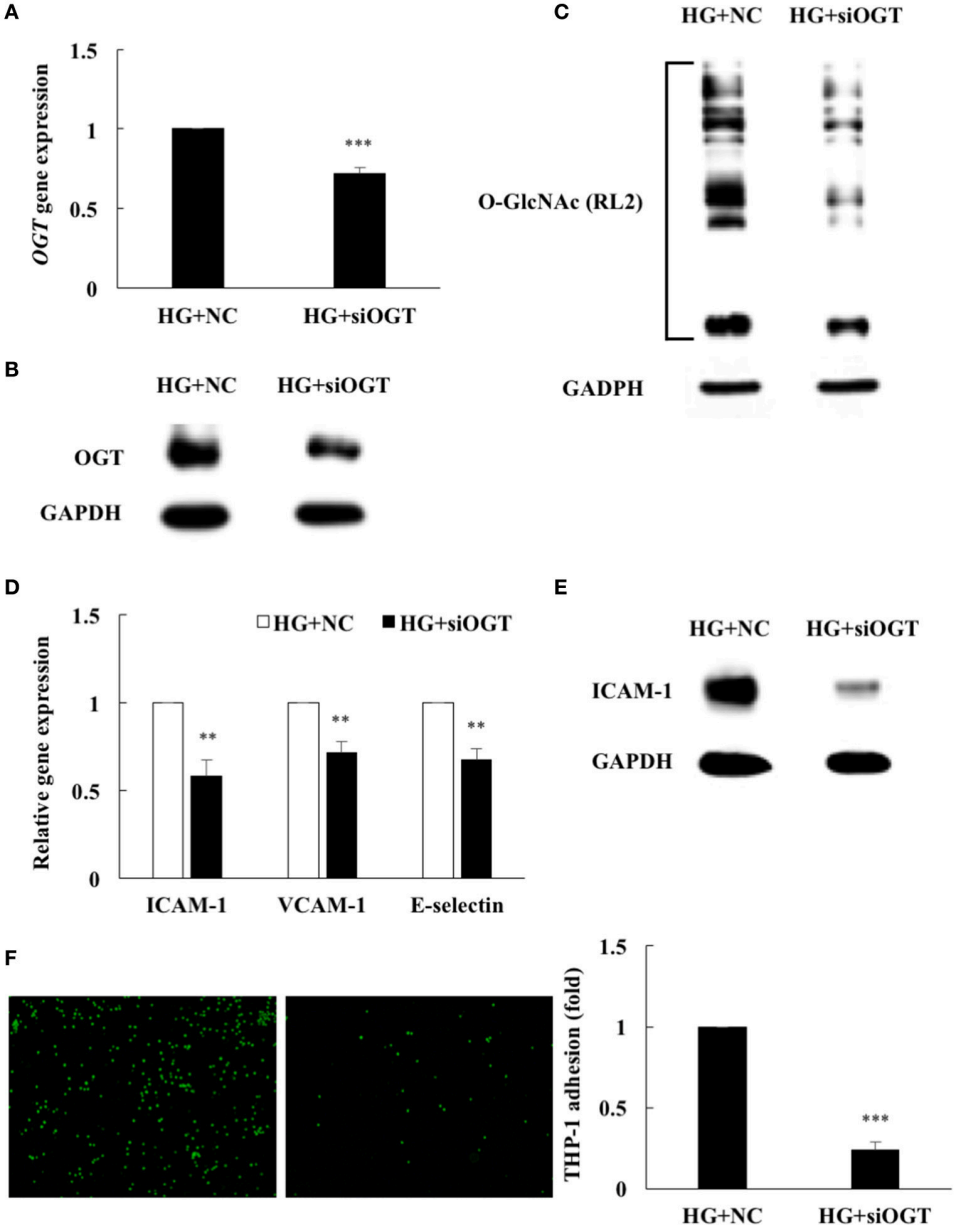
HG-induced endothelial protein O-GlcNAcylation and inflammation were reduced by OGT siRNA transfection. **(A)** OGT gene expression levels were reduced to 72% of the control level by OGT siRNA in HG-stimulated HAECs (HG+siOGT) *N* = 5. ***p < 0.001 compared with HG-treated HAECs transfected with negative control (HG+NC). **(B)** OGT protein expression levels were significantly reduced by OGT siRNA in HG-stimulated HAECs, as compared with HG+NC. The blot is representative of three independent experiments. **(C)** Protein *O*-GlcNAcylation levels were significantly reduced by OGT siRNA in the HG-stimulated HAECs, as compared with HG+NC. The blot is representative of three independent experiments. **(D)** siOGT transfection inhibited HG-induced endothelial ICAM-1 mRNA expression to 58%, VCAM-1 mRNA expression to 71%, E-selectin mRNA expression to 67% of the control level in the HG-stimulated HAECs *N* = 5. **p < 0.01 compared with HG+NC. **(E)** ICAM-1 expression levels were significantly reduced by OGT siRNA in HG-stimulated HAECs, as compared with HG+NC. The blot is representative of three independent experiments. **(F)** siOGT transfection inhibited HG-induced THP-1 adhesion to 24% of the control level in the HG-stimulated HAECs *N* = 3. ***p < 0.001 compared with HG+NC.

To determine whether increased OGT expression was associated with HG-induced protein *O*-GlcNAcylation and inflammation, an OGT-specific siRNA transfection experiment was performed. As shown in Figure 4A, OGT-specific siRNA transfection caused a significant decrease in OGT mRNA levels—to 72% of the levels in the cells transfected with the scrambled negative control. Transfection of OGT siRNA also significantly attenuated OGT protein expression (Figure 4B) and was associated with decreased protein *O*-GlcNAcylation (Figure 4C), confirming that HG-induced endothelial *O*-GlcNAcylation was mediated partially through OGT. To determine whether reduced OGT and protein *O*-GlcNAcylation expression were associated with reduced endothelial inflammation in OGT-depleted HG-stimulated HAECs, we measured the ICAM-1, VCAM-1, and E-selectin gene expression levels, ICAM-1 expression, and THP-1 monocyte adhesion. Similar to the effects of the miR-200a/200b mimics, OGT siRNA transfection caused a significant decrease in ICAM-1, VCAM-1, and E-selectin mRNA levels—to 58, 71, and 67%, respectively, of that in the scrambled negative control (Figure 4D). ICAM-1 expression also significantly decreased following transfection of OGT siRNA in the HG-stimulated HAECs (Figure 4E) and was associated with decreased THP-1 cell adhesion to the HAECs—to 24% of the adhesion levels in the scrambled negative control group (Figure 4F)—indicating that HG-induced endothelial inflammation is mediated in part through OGT.

### miR-200a/200b mimics decreased endothelial OGT and ICAM-1 expression in type 2 diabetic mice

**Figure 5:**
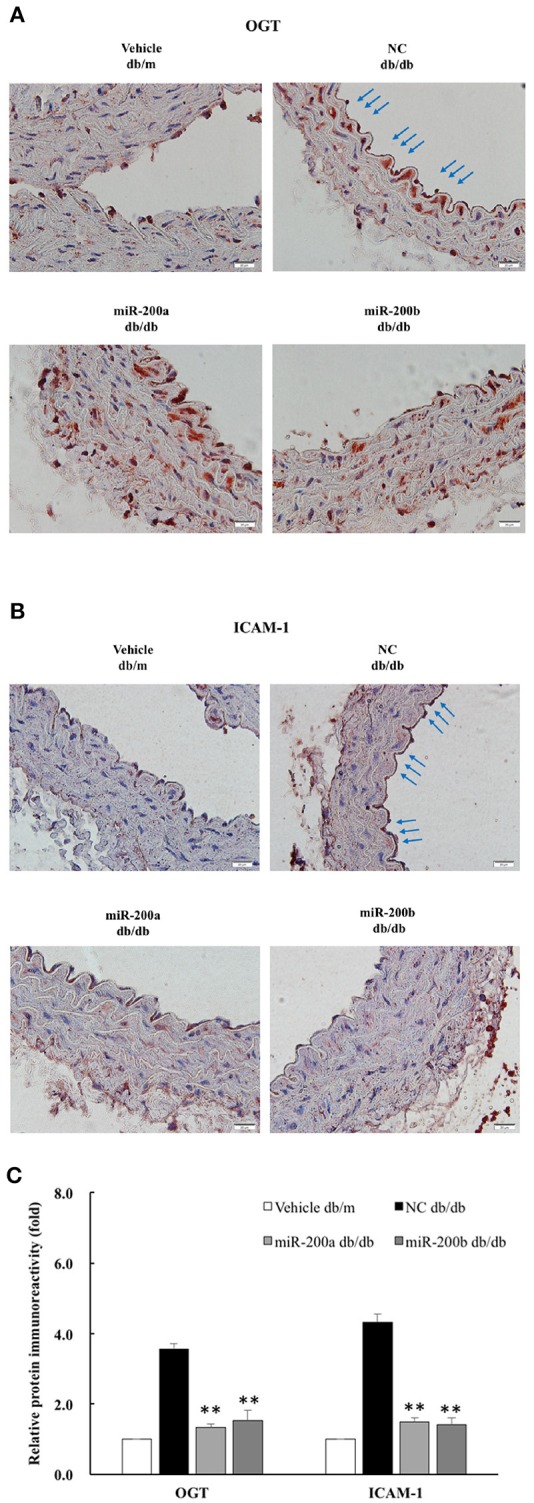
miR-200a/200b mimic decreased endothelial OGT and ICAM-1 expression in type 2 db/db diabetic mice. Immunohistochemistry staining of **(A)** OGT and **(B)** ICAMI-1 in the thoracic aorta tissue. Representative images showing that the immunoreactivities of endothelial OGT and ICAM-1 in the aortic endothelial layers (blue color as arrow) were decreased in the miR-200a/200b mimic-treated db/db mice, as compared with the negative control (NC)-treated db/db mice. db/m mice: non-diabetic control mice. *N* = 3 per group. Scale bar = 20 μm. **(C)** Immunoreactivity signals in endothelial layers from four treatment groups were quantified for all immunohistochemistry images. **p < 0.01 compared to the db/db (NC) group.

**Figure 6:**
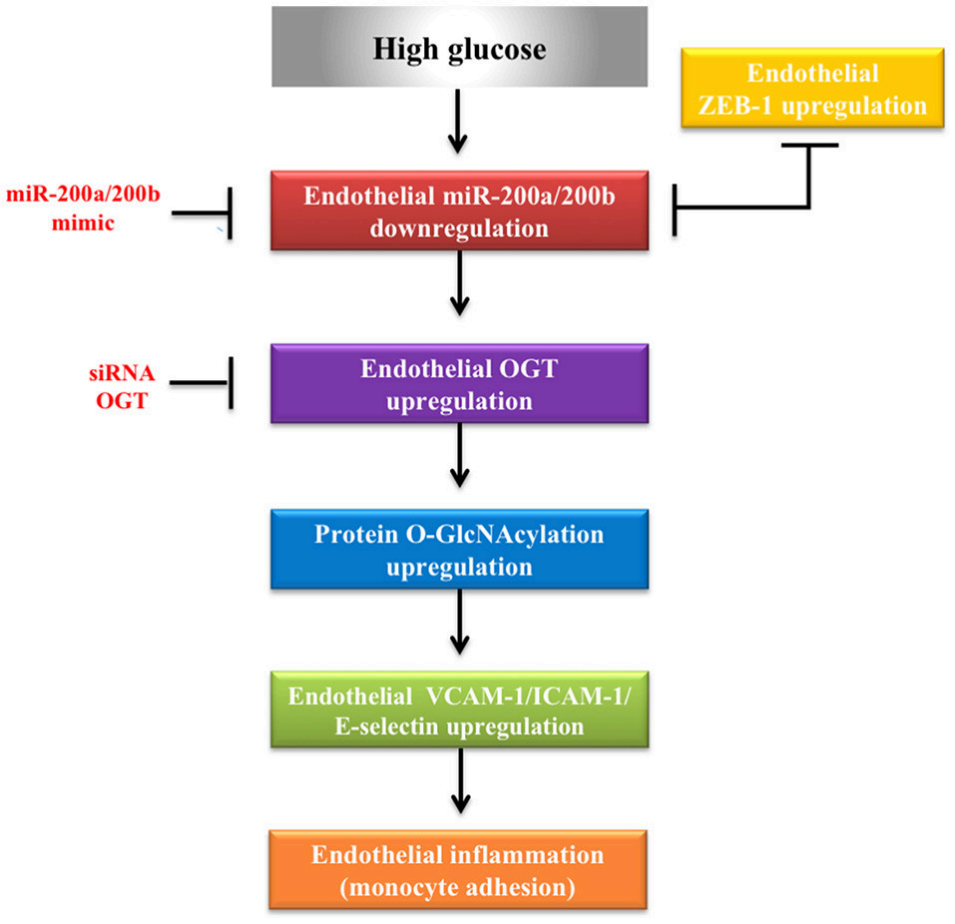
Proposed role of miR-200a/200b in regulating HG-induced endothelial protein *O*-GlcNAcylation and inflammation via OGT.

To examine the effect of miR-200a/200b mimics on the expression of endothelial OGT and ICAM-1 in db/db type 2 diabetic mice, we performed IHC staining of the aortic tissues. As shown in Figures 5A,B, the immunoreactivities of OGT and ICAM-1 in the aortic endothelial layers were substantially downregulated in the miR-200a/200b mimic-treated db/db mice than in the negative control-treated db/db mice. In the endothelial layers, quantification of immunoreactivity signals showed that OGT expression was decreased significantly—to 0.37- and 0.43-fold in the db/db (miR-200a mimic) and db/db (miR-200b mimic) groups compared with that in the db/db (negative control) group. ICAM-1 expression in the endothelial layers was also decreased significantly—to 0.34- and 0.33-fold in the db/db (miR-200a mimic) and db/db (miR-200b mimic) groups compared with that in the db/db (negative control) group (Figure 5C). This suggests that miR-200a/200b mimics may play a therapeutic role by decreasing diabetes-induced endothelial inflammation through the downregulation of both OGT and ICAM-1. The potential mechanisms of miR-200a/200b in regulating HG-induced endothelial OGT expression, protein O-GlcNAcylation, and inflammation is illustrated in Figure 6.

## Discussion

The present study demonstrates that HG induces OGT expression, protein *O*-GlcNAcylation levels, and endothelial inflammation in the HAECs by downregulating miR-200a/200b expression. Increased OGT expression and protein O-GlcNAcylation showed pro-inflammatory effects in the HG-treated HAECs, representing one of the important mechanisms sustaining endothelial inflammation in diabetes. Both cellular and animal experiments revealed miR-200a/200b as a potential therapeutic target for treating vascular complications in diabetes.

The role of *O*-GlcNAcylation in regulating cellular inflammation is complex and depends on the stress type and stress duration. Chen et al. reported that glucosamine and PUGNAc (an OGA inhibitor) inhibited ICAM-1 expression, cell adhesion, and nuclear factor-κB activity in tumor necrosis factor-α-stimulated human pigment epithelial cells (Chen et al., 2012). Hilgers et al. reported that glucosamine and Thiamet-G (an OGA inhibitor) suppressed tumor necrosis factor-α-induced hypocontractility and endothelial dysfunction by inhibiting tumor necrosis factor-α-induced inducible nitric oxidase synthase expression and peroxynitrite production in the rat aortic ring (Hilgers et al., 2012). Xin et al. reported that glucosamine treatment for 14 days inhibited neointimal formation in the injured carotid artery in a rat acute balloon injury model (Xing et al., 2008). These studies support that *O*-GlcNAcylation has a protective role in acute stress.

In contrast, *O*-GlcNAcylation has been implicated in the development of cardiovascular complications in diabetes-related studies (Issad et al., 2010; Lima et al., 2012). James et al. reported that nuclear factor-κB-dependent promoter activation occurred because of increased flux in HBP in rat mesangial cells (James et al., 2002). Du et al. reported that HG-induced activation of HBP increased *O*-GlcNAcylation of Sp-1, Sp-1 transactivation, and Sp-1-dependent plasminogen activator inhibitor-1 and transforming growth factor-β gene expression in bovine aortic endothelial cells (Du et al., 2000). Federici et al. reported that HG and glucosamine-induced *O*-GlcNAcylation impaired insulin signaling by downregulating endothelial nitric oxide synthase phosphorylation and activity in human coronary endothelial cells (Federici et al., 2002). Makino et al. reported that OGA overexpression significantly decreased endothelial protein *O*-GlcNAcylation, and simultaneously increased left ventricle capillary density and restored the endothelium-dependent relaxation in streptozotocin-induced type 1 diabetic mice (Makino et al., 2015). This study demonstrates that HG induced the expression of the essential *O*-GlcNAcylation enzyme OGT. Importantly, we observed a crucial role for increased OGT expression and protein *O*-GlcNAcylation in the regulation of endothelial inflammation, as HG-induced ICAM-1, VCAM-1, and E-selectin mRNA expression; ICAM-1 expression; and THP-1 monocyte adhesion were reduced after OGT depletion by siRNA. Interestingly, our data showed that HG increased OGA mRNA. In addition, the miR-200b mimic not only decreased the expression levels of protein *O*-GlcNAcylation, but also decreased OCA mRNA. Previously, Zhang et al. reported that OGA mRNA and protein expression is sensitive to cellular protein *O*-GlcNAcylation levels (Zhang et al., 2014). Our data also suggest that OGA plays a significant role in maintaining protein *O*-GlcNAcylation homeostasis by responding to the cellular protein *O*-GlcNAcylation levels (i.e., increase protein *O*-GlcNAcylation causing increase OGA expression and vice versa).

In this study, web-based bioinformatics analysis using miRanda-mirSVR and miRDB database revealed that the members of the miR-200 functional group 1, but not of group 2, may bind to the 3′-UTR of human OGT mRNA. However, the miRanda-mirSVR database identified all five miR-200 family members as potential binding partners of the mouse Ogt 3′-UTR. Based on manual sequence alignment, both human OGT and mouse Ogt 3′-UTRs share an identical nucleotide sequence (5′-AGUAUU-3′) that is complementary to the seed sequence of miR-200 functional group 2, suggesting that human OGT 3′-UTR also binds to miR-200b/200c/429, similar to mouse Ogt. Alternatively, G:U wobble pairing may explain the binding of miR-200b (seed sequence: 3′-GUCAUAA-5′) to the miR-200a binding site on OGT 3′-UTR (5′-CAGUGUU-3′), as Doench et al. reported that thermodynamically favorable G:U pairing in the seed region can retain significant gene repressive effects (Doench and Sharp, 2004). In this study, miR-200a/200b mimics regulated OGT gene and protein expression and protein *O*-GlcNAcylation. In addition, a luciferase reporter assay confirmed that miR-200a/200b mimics directly interact with the 3′-UTR of OGT mRNA. These findings support that the miR-200a::OGT and miR-200b::OGT interactions are functional. While we did not investigate the role of miR-141 and miR-200c/429 in regulating OGT expression, it is possible that all members of the miR-200 family can regulate OGT expression, as they have the same seed sequence as either miR-200a or miR-200b.

Previously, Liu et al. reported that ICAM-1 is a direct target of miR-141 in the human umbilical endothelial cells (Liu et al., 2015). They further examined the role of an intravenous miR-141 mimic in a mouse model of myocardial ischemia-reperfusion injury and found that the miR-141 mimic attenuated myocardial ICAM-1 levels and infarction size (Liu et al., 2015). Although we did not examine the repressive effect of miR-141 on ICAM-1 expression, we found that ICAM-1 gene and protein expression and monocyte adhesion were significantly downregulated by the miR-200a mimic. Given that miR-200a and miR-141 have the same seed sequence, miR-200a may also regulate ICAM-1 expression in the HG-stimulated HAECs. In addition, similar to OGT 3′-UTR, *in silico* analysis (www.microrna.org) did not identify functional group 2 members as targeting ICAM-1; however, we found that the miR-200b mimic significantly decreased ICAM-1 expression in HAECs. This suggests that either miR-200b can bind the miR-200a binding site of the ICAM-1 3′-UTR through G:U wobble binding, or ICAM-1 was indirectly repressed by miR-200b. Interestingly, the miR-200 family members were not predicted to bind to the mouse ICAM-1 3′-UTR; however, our results show that miR-200a/200b mimics significantly attenuated endothelial ICAM-1 expression, suggesting that ICAM-1 expression was indirectly regulated by miR-200a/200b mimics in the db/db diabetic mice. Based on OGT siRNA data, the ability of miR-200a/200b mimics to downregulate OGT expression and *O*-GlcNAcylation levels may act as a possible mechanism to attenuate hyperglycemia-induced endothelial inflammation by regulating nuclear factor-kB activity (James et al., 2002; Yang et al., 2008; Allison et al., 2012).

There is conflicting evidence regarding the regulatory role of miR-200 family members in diabetes. The miR-200 family has been reported to have a detrimental effect. Reddy et al. reported that vascular smooth muscle cells and aortic tissue from db/db mice showed increased miR-200b/429 expression, and an miR-200b mimic increased monocyte binding to db/+ vascular smooth muscle cells, whereas an miR-200b inhibitor decreased monocyte binding in the db/db vascular smooth muscle cells (Reddy et al., 2012). Belgardt et al. reported that β-cell specific overexpression of miR-200 family induced β-cell apoptosis and type 2 diabetes, whereas miR-200 family knock-out reduced β-cell apoptosis and improved type 2 diabetes symptoms (Belgardt et al., 2015). Zhang et al. reported that miR-200c expression was increased in the diabetic mouse aorta and HG-stimulated mouse aortic endothelial cells. Moreover, they noted that a miR-200c mimic impaired endothelial-dependent relaxation in the non-diabetic mouse aorta, whereas an miR-200c inhibitor enhanced endothelial-dependent relaxation in the diabetic db/db mice (Zhang et al., 2016).

In contrast, the miR-200 family has also been reported to have protective effects. Wei et al. reported that tail-vein injection of anti-miR-200a in streptozotocin-induced diabetic mice exacerbated cortical and glomerular fibrosis and increased urinary albumin excretion (Wei et al., 2014). Vascular endothelial growth factor plays an important role in the pathogenesis of diabetic retinopathy (Chiefari et al., 2016). McArthur et al. observed miR-200b downregulation and vascular endothelial growth factor (a target of miR-200b) upregulation in streptozotocin-induced diabetic rat retinas. Furthermore, intraocular injection of an miR-200b mimic prevented an increase in vascular endothelial growth factor expression and vascular permeability. Importantly, human retinal tissue from diabetic enucleated eyes showed lower miR-200b expression than non-diabetic retina (McArthur et al., 2011). Mitra et al. also reported the application of miR-200b nanoparticles for treating diabetic retinopathy in mice (Mitra et al., 2016). These findings suggest that the miR-200 family has cytoprotective roles, consistent with its role as a tumor suppressor. Previous studies reported that *O*-GlcNAcylation may be either beneficial or harmful, depending on the experimental design (e.g., type of species or tissue, acute vs. chronic stress, diabetic vs. non-diabetic samples), suggesting that the miR-200 family has various roles in inflammation-associated diseases, including diabetes. However, this study confirmed that the miR-200 family is dysregulated in HG-stimulated HAECs, and both *in vitro* and *in vivo* experiments revealed that miR-200a/200b have therapeutic potential in treating diabetic vascular disease.

## Author contributions

H-JW, C-TP, and W-YL conceived the project. H-JW and W-YL wrote the manuscript. H-JW, C-TP, and W-YL provided funding. H-JW, W-KY, W-YP, and W-YL performed critical experiments. W-YL and H-JW supervised the study.

### Conflict of interest statement

The authors declare that the research was conducted in the absence of any commercial or financial relationships that could be construed as a potential conflict of interest.
