# Corrigendum: MicroRNA-200a/200b Modulate High Glucose-Induced Endothelial Inflammation by Targeting *O*-linked *N*-Acetylglucosamine Transferase Expression

**DOI:** 10.3389/fphys.2018.00786

**Published:** 2018-06-19

**Authors:** Wan-Yu Lo, Wen-Kai Yang, Ching-Tien Peng, Wan-Yu Pai, Huang-Joe Wang

**Affiliations:** ^1^Cardiovascular and Translational Medicine Laboratory, Department of Biotechnology, Hungkuang University, Taichung, Taiwan; ^2^Bachelor Degree Program in Animal Healthcare, Hungkuang University, Taichung, Taiwan; ^3^Department of Life Sciences, National Chung Hsing University, Taichung, Taiwan; ^4^Department of Pediatrics, Children's Hospital, China Medical University and Hospital, Taichung, Taiwan; ^5^Department of Bioscience and Biotechnology and Center of Excellence for the Oceans, National Taiwan Ocean University, Keelung, Taiwan; ^6^School of Medicine, China Medical University, Taichung, Taiwan; ^7^Cardiovascular Research Laboratory, Division of Cardiovascular Medicine, Department of Internal Medicine, China Medical University and Hospital, Taichung, Taiwan

**Keywords:** diabetes, high glucose, endothelial inflammation, *O*-linked *N*-acetylglucosamine transferase, microRNA-200a/200b

In the published article, there was an error in affiliation 2. Instead of “Program in Animal Healthcare, Hungkuang University, Taichung, Taiwan”, it should be “Bachelor Degree Program in Animal Healthcare, Hungkuang University, Taichung, Taiwan”. The authors apologize for this error and state that this does not change the scientific conclusions of the article in any way.

The original article has been updated.

## Conflict of interest statement

The authors declare that the research was conducted in the absence of any commercial or financial relationships that could be construed as a potential conflict of interest.

