# Supplementary material for: MicroRNA-200a/200b Modulate High Glucose-Induced Endothelial Inflammation by Targeting O-linked N-Acetylglucosamine Transferase Expression
Source: Front Physiol. 2018 Apr 18;9:355. doi: 10.3389/fphys.2018.00355 (PMC5915961; doi:10.3389/fphys.2018.00355)

MicroRNA-200a/200b Modulate High Glucose-Induced Endothelial Inflammation by Targeting *O*-linked *N*-Acetylglucosamine Transferase Expression

Wan-Yu Lo; Wen-Kai Yang; Ching-Tien Peng; Wan-Yu Pai; Huang-Joe Wang*

**Supplement Data 1.** Primer set list for mRNAs	
**GAPDH**	Forward primer: 5'-CTCTGCTCCTCCTGTTCGAC-3'<br>Reverse primer: 5'-ACGACCAAATCCGTTGACTC-3'
**OGT**	Forward primer: 5'-GCAGCAGGACCAATTACCTC-3'<br>Reverse primer: 5'-GCATACGTTTCGTTGGTTCTG-3'
**ICAM-1**	Forward primer: 5’-CCTTCCTCACCGTGTACTGG-3’<br>Reverse primer: 5’-AGCGTAGGGTAAGGTTCTTGC-3’
**VCAM-1**	Forward primer: 5’-TGCACAGTGACTTGTGGACAT-3’<br>Reverse primer: 5’-CCACTCATCTCGATTTCTGGA-3’
**E-selectin**	Forward primer: 5ˊ-ACCAGCCCAGGTTGAATG-3ˊ<br>Reverse primer: 5ˊ-GGTTGGACAAGGCTGTGC-3ˊ
**ZEB-1**	Forward primer：5ˊ-CGAAACGCGAGGTTTTGTA-3ˊ<br>Reverse primer：5ˊ-TCTAGACAGGAAATCCCACACA-3ˊ
**OGA**	Forward primer：5ˊ-TGGTCTAGCAGGAGAGTTCCA-3ˊ<br>Reverse primer：5ˊ-AAACTTTGGAGGTAGGAGTCAGTG-3ˊ

Supplemental data 2. The inserted target sequence of pmirGLO-OGT-3'UTR. The potential miR-200a binding site and miR-200b binding site are labeled with red and green color, respectively.

1………………………………………………………………………………...50
ATGACTCAAG ATTTTTTCTG GTCCATTTCC CATTTCCTTT TCTTCCCTGA

51………………………………………………………………………………100
CCCCCATACC CTCACCCTTA AAATTCTCCT GTAACTCAAC TAACAAAATC

101……………………………………………………………………………......150
AAGCCTGATT CAAAACATCC TAGGGTGTTT TAAACACACC ATCTGGTGCC

151……………………………………………………………………………......200
AAATGAAGAT TTTTAGGAGT GATTACTAAT TATCAAGGGC ACAGTTGTGG

201……………………………………………………………………………......250
TACTGTCATT GATAATAATA TAGTTTTTTT TTTTTTCCTA ATTTTGACCT

251……………………………………………………………………………......300
GTTTCACCAG TGTTTTACCC TTGACTGCCC CTTCTATGCT GCTTCCAAAA

301……………………………………………………………………………...350
GTGATAGTGT GTGTAAGATT TTTACCTTCC TTTCTAAAGT TTTTTTTTTT

351……………………………………………………………………………...400
TTTTTTAAGT GAGTCCTGTT CTTCCTATTT CTTTCAGCAG AAATGAAATC

401……………………………………………………………………………...450
CCAGGTAAGT ATAAGTATTC AAGTATTTGA TCAGTAAGTC ACAGTTATCT

451……………………………………………………………………………...500
CCAGTGCATT AAATAACCTT CATCAAGAAA TAGGTTATAG GTAAAATCTC

501……………………………………………………………………………………..550

TGAAGGATCA TCTATGTATT CAAGTAATTA TTTTTTAGAT AATAACTGTC

551……………………………………………………………………………………..600

TTCTGGACTT GGTCTTGAAG TCTGTACAGA TTCAGCCTCA GTAGTAGCGA

Supplemental Data 3. Endothelial OGA mRNA expression was significantly increased by HG. N=3.

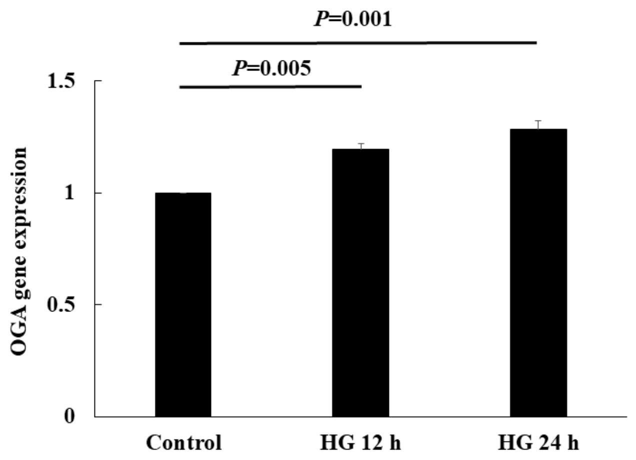

Supplemental Data 4. Transfection of miR-200a/200b mimics inhibited HG-induced endothelial OGA mRNA expression to 95% and 91% of the control levels, respectively. N=4.

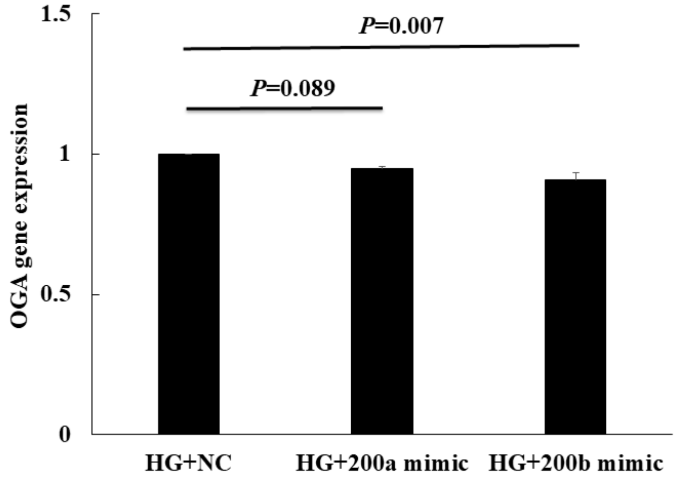

Supplement: Supplementary file 1 [file DataSheet1.PDF]
